# Ensuring trustworthy AI assisted guideline development for clinical practice

**DOI:** 10.1038/s41746-026-03098-z

**Published:** 2026-08-27

**Authors:** Quan Zhang, Yuanyuan Fu

**Affiliations:** 1 Department of Quantitative Health Sciences, John A. Burns School of Medicine, University of Hawaiʻi, Honolulu, HI USA; 2 Department of Economics, College of Social Sciences, and University of Hawaiʻi Economic Research Organization (UHERO), University of Hawaiʻi, Honolulu, HI USA; 3 Cancer Biology Program, University of Hawaiʻi Cancer Center, Honolulu, HI USA; 4 Medical AI Core, Pacific Center for Artificial Intelligence and Data Science in Medicine, University of Hawaiʻi at Manoa, Honolulu, HI USA

**Keywords:** Computational biology and bioinformatics, Health care, Mathematics and computing, Medical research, Scientific community

## Abstract

Li et al. introduce Quicker, an agentic large language model system that drafts clinical guideline recommendations through a GRADE-based workflow. Building on gaps in the authors’ own evaluation, we propose safeguards for trustworthy use: verifiable, design-aware evidence bundles with explicit scope; calibrated, uncertainty-aware deferral; targeted human validation and modular quality gates; and governance against contamination and over-reliance. Trustworthiness, however, is necessary but not sufficient for clinician trust.

Li et al. report “Quicker,” an agentic large language model (LLM) system that operationalizes the end-to-end workflow of evidence-based clinical recommendation development, from question decomposition and literature search to study selection, evidence assessment, and recommendation drafting. In system-level testing with a single reviewer, Quicker formulated individual recommendations in 20–40 min, compressing steps of a process that commonly takes months^1^. Quicker combines multi-stage prompting, an iterative literature-search agent connected to the PubMed API, and retrieval-augmented generation (RAG) to ground question decomposition, full-text assessment, and evidence assessment in retrieved sources; during evidence assessment, it applies the GRADE framework, with the LLM performing risk-of-bias assessment and compiling evidence profiles^1^. The accompanying Q2CRBench-3 benchmark, derived from three guideline-development datasets, includes about 26,200 retrieved records, of which 99.49% are excluded during screening; this figure illustrates the screening bottleneck that often constrains guideline updates^1^. Together, these contributions suggest that evidence synthesis and draft recommendations could be produced on demand, enabling more frequent and responsive guideline maintenance.

The translational challenge, however, is not speed; it is trust. Clinical guidelines influence care pathways, quality metrics, and reimbursement decisions, so an overconfident or incorrectly grounded recommendation can propagate widely. An evaluation framework for clinical LLM use argues that safe deployment requires task-specific testing of accuracy, robustness, interaction risks, and deferral behavior, not only agreement with a reference output, and shows that fluent answers can still contain errors or unsupported inferences when the evidence context is incomplete or ambiguous^2^. Because Quicker relies on GPT-4o for most tasks, the limitations documented for that model are directly relevant^3^. Building on these insights, and on the authors’ own caution that Quicker outputs require careful review^1^, we propose several implementation-focused safeguards to strengthen reliability and clinical acceptability.

## Verifiable evidence bundles

AI outputs should be delivered as verifiable evidence bundles rather than free-text recommendations. Quicker’s staged pipeline makes provenance achievable, because it already retrieves, screens, and assesses evidence before drafting conclusions^1^. We recommend that each draft recommendation include (i) traceable citations to the exact included studies; (ii) a structured evidence table (PICO elements, study design, per-outcome effect estimates, a risk-of-bias or certainty rating, and key limitations) with links to the supporting passages used for extraction; (iii) an explicit mapping between evidence items and each statement in the rationale; and (iv) a concise scope-and-limitations statement documenting the databases and date ranges searched, the language and publication-type filters applied, and known coverage gaps, such as paywalled full texts, trial registries, preprints, and non-English sources. Such a statement matters because Quicker’s screening sensitivity varied across datasets, falling to about 0.79 in one dataset under the basic method^1^, so users can see the boundaries of the evidence actually retrieved. Recent clinical development guidelines for RAG in biomedicine emphasize that disciplined retrieval design and transparent citation practices are critical for improving reliability and enabling auditability^4^. Operationally, this evidence-bundle design would let guideline panelists verify core claims rapidly, see where evidence is thin or where search coverage is incomplete, and correct extraction errors without rereading the full corpus.

## Actionable uncertainty and calibrated deferral

Quicker should make uncertainty visible and actionable at the point of recommendation. Even with RAG, models can overgeneralize from sparse evidence, smooth over heterogeneity, or express unwarranted confidence when data are weak or conflicting^2^. We encourage an uncertainty-aware output format that separates evidence statements (what studies show) from normative choices (what should be recommended). It should report two distinct quantities: the GRADE certainty of the evidence for each critical outcome, and the model’s own confidence in its extraction and synthesis. These can diverge, so deferral should be triggered by low model confidence rather than by evidential certainty alone. Because verbalized LLM confidence is often poorly calibrated, this signal should itself be validated rather than treated as a free parameter, for example, through self-consistency across decodes, disagreement across the models Quicker was run on (GPT-4o, Claude 3.5 Sonnet, and DeepSeek-v3), or calibration against held-out guideline-panel labels^2^. Systematic prompt testing and versioning should likewise be part of deployment, given evidence that prompt design affects reliability in guideline contexts^5^. At the same time, deferral is not costless. A system tuned to abstain too readily could withhold well-supported recommendations and delay care, suppressing valid patient-care recommendations and reproducing the inefficiency the pipeline aims to reduce. This risk is not hypothetical: Li et al. report that Quicker’s autonomous, domain-by-domain downgrading sometimes over-downgraded evidence^1^. Deferral thresholds should therefore be explicit, tunable, and calibrated against clinical consequences, and evaluation should track both error directions, namely recommendations issued on weak evidence (false confidence) and valid recommendations withheld (over-deferral). The goal is calibrated caution, not blanket conservatism.

## Design-aware evidence assessment

The evidence-assessment stage should be made design-aware. Quicker already applies GRADE and has the LLM perform risk-of-bias assessment, but that assessment was built and validated around randomized controlled trials, using a trial-oriented instrument (RoB 2)^1^. Guideline questions, however, frequently rest on non-randomized and observational evidence, particularly for questions of harm and for rare conditions or settings where randomized trials are absent or unethical. That Quicker misclassified an open-label pilot RCT as an observational study^1^ shows that study design cannot be assumed and must be handled explicitly. For non-randomized studies of interventions, risk of bias should be appraised with a design-appropriate tool such as ROBINS-I rather than an instrument built for trials, with the appropriate tool selected by question type^6,7^. Certainty rating should then follow one internally consistent GRADE convention rather than stacking two: either begin non-randomized evidence at low certainty under the traditional approach, or, when appraising with ROBINS-I, begin at high certainty and rate down through the ROBINS-I domains, which avoids double-counting confounding^8^. Finally, outputs should flag when a recommendation depends primarily on observational evidence, so that neither the system nor its users treats randomized and observational findings as interchangeable.

## Standardized human validation

The human-in-the-loop step should be formalized as a lightweight but standardized validation protocol, concentrated where Quicker is known to be weakest. Li et al. report that Quicker’s numerical data extraction reached only about 72% precision, that its risk-of-bias ratings agreed poorly with guideline panels, and that it overlooked selective reporting and struggled with missing-data bias^1^. These are exactly the steps a short checklist should target: confirm eligibility for a sample of borderline studies; verify extraction of primary outcomes and key effect estimates for high-impact trials; review risk-of-bias judgments with attention to selective reporting and missing data; and confirm that each recommendation is consistent with its linked evidence bundle. Prior work integrating LLMs into systematic reviews shows where human verification is most needed, including error-sensitive risk-of-bias steps that can alter conclusions^6^, and that automation raises throughput but still benefits from targeted checks at high-impact points^9^. Because full re-review would negate the speed advantage, this protocol is deliberately a risk-stratified sample rather than comprehensive re-checking; the residual efficiency gain over a months-long process is real but smaller than the raw 20–40 min figure implies.

## Broader prospective validation

Broader validation is needed to establish generalizability and to detect bias before wide deployment. Although Q2CRBench-3 spans multiple diseases^1^, guideline development differs across specialties, populations, study designs, and outcome types, and the system-level test was conducted with a single reviewer on RCT evidence^1^. Real-world workflows also face heterogeneous evidence access, including paywalled full texts, registries, and preprints; the authors note that validation was conducted primarily on PubMed because of access constraints, and that users may need to upload additional evidence manually^1^. We therefore suggest prospective pilots with guideline committees in several specialties and settings, using pre-specified endpoints such as screening sensitivity, extraction error rates for key outcomes, stability of recommendations across model versions, and subgroup coverage in the underlying evidence. Benchmarking studies in evidence-based medicine have found substantial task-to-task variability in LLM performance, which reinforces the need for context-specific validation rather than general claims of capability^10^. Domain-specific calibration approaches for guideline interpretation have also been proposed, indicating that retrieval design and local adaptation can materially influence accuracy^11^.

## Modular quality gates

Quicker should be evaluated and governed as a modular evidence-synthesis pipeline, not as a monolithic generator. A growing literature maps how LLMs can assist distinct systematic-review components, such as screening^9^ and risk-of-bias assessment^6^, while also documenting limitations that argue for component-level evaluation and error tracing^12^. Quicker’s decomposition makes stage-specific quality gates feasible. Li et al. already report stage metrics such as screening sensitivity^1^; the incremental proposal is to convert these from one-time benchmark reporting into prospectively enforced, pre-registered thresholds with fail-closed behavior and per-deployment monitoring, for example, recall floors for screening, extraction-fidelity checks, and consistency checks between evidence tables and drafted rationales. This modular governance aligns with benchmarking work in evidence-based medicine^10^ and complements recent systems that accelerate clinical evidence synthesis with LLMs through staged pipelines and explicit intermediate artifacts^13^. Looking forward, the vision of automated, living, and interactive evidence synthesis implies that reliable pipelines will need continuous monitoring and updating, not one-time validation^14^.

## Governance against contamination and over-reliance

Transparency and data governance should be designed to prevent downstream contamination and loss of accountability. Li et al. themselves highlight risks of benchmark leakage and deployment-related contamination, assess memorization and overlap, and provide a canary string to support traceability^1^. Scholarship on user trust further warns that self-referential learning loops could amplify bias and contribute to deskilling if clinicians become over-reliant on machine-generated recommendations^15^. We recommend extending Quicker’s governance with (i) clear labeling of AI-assisted drafts and their model and version identifiers; (ii) retention of structured logs (retrieval results, inclusion decisions, extraction tables, and validation actions) to enable audit and post hoc error analysis; and (iii) explicit policies preventing unreviewed AI-generated guideline text from being treated as evidence or reused as training data.

## Toward trustworthy adoption

In summary, Li et al.’s evaluation indicates that LLMs can substantially accelerate evidence synthesis and recommendation drafting^1^. Translating this efficiency into safe clinical impact will require outputs that are verifiable, design-aware, and auditable, together with calibrated deferral, targeted human validation, and governance against contamination and over-reliance^2–15^. Crucially, certainty of evidence is only one input to a recommendation. The move from evidence to recommendation requires the value judgments formalized in the GRADE Evidence-to-Decision framework (benefits and harms, patient values, resource use, equity, feasibility), which the system should surface inputs for but a human panel should resolve^1,8^.

We close with a distinction that bears on adoption. The safeguards proposed here target the trustworthiness of Quicker’s outputs, an evidentiary property of the system, whereas clinician trust is a distinct, relational attitude toward it. Trustworthiness can warrant trust, but it does not by itself produce it; appropriate trust also depends on clinicians’ direct experience with the system, on institutional adoption processes, and on clear communication about when the system is and is not reliable. Establishing trustworthy pipelines is therefore necessary but not sufficient for warranted trust and safe uptake, and overtrust in fluent yet unverified outputs remains a distinct risk that governance and training must actively manage^15^.

We also note the limitations of this commentary. Our safeguards are conceptual proposals informed by the published description of Quicker and by the surrounding literature; we did not have access to the system and did not test the proposals empirically, nor do we present a worked demonstration. Each safeguard also carries costs, including added reviewer workload, computational overhead, and potential latency, that must be weighed against its benefits, and the safeguards themselves warrant prospective evaluation. We therefore present them as hypotheses for implementation and study rather than as validated requirements. Taken together, these steps can help bridge the gap between fast AI-generated drafts and trustworthy guideline adoption in routine clinical practice.

## Data Availability

Not applicable. This Matters Arising reports no new data generation or analysis.
